# Revision of the rhinoceros beetle genus *Oryctophileurus* Kolbe with description of a new species, the male of *O. varicosus* Prell, and notes on biogeography (Scarabaeoidea, Dynastinae, Phileurini)

**DOI:** 10.3897/zookeys.346.6114

**Published:** 2013-11-01

**Authors:** Robert Perger, Paschoal Coelho Grossi

**Affiliations:** 1 Colección Boliviana de Fauna. Casilla 10077, Correo Central. La Paz, Bolivia; 2 Universidade Federal do Paraná, Centro Politécnico, Departamento de Zoologia, Caixa Postal 19007, CEP, 81531-980, Curitiba, Paraná, Brazil; 3 Departamento de Biologia e Zoologia, Instituto de Biociências, Universidade Federal de Mato Grosso – UFMT, Av. Fernando Corrêa da Costa, 2367, Boa Esperança, CEP 78060-900, Cuiabá, MT, Brazil

**Keywords:** Andes, Melolonthidae, Neotropical, relictual species, South America, Tucuman-Bolivian forest

## Abstract

The genus *Oryctophileurus* is reviewed and its validity is supported by a combination of the following apomorphic characters: a single cephalic horn with lateral carina, pronotal cavity with ocellate punctures and two teeth or tubercles close behind the anterior pronotal margin. The male of *Oryctophileurus varicosus* Prell, 1934, is described for the first time. A new species, *Oryctophileurus guerrai* Perger & Grossi **sp. n.**, from subhumid Tucuman-Bolivian forest in the Southern Bolivian Andes is described. The new species is distinguished from its closest relative, *O. armicollis* Prell, 1911, by a narrower distance between the inner teeth of the dorsal pronotal protuberances and a reduced area of weakly developed ocellate punctures above the posterolateral pronotal margin. The occurrence of *Oryctophileurus* species in areas of endemism along the eastern slope of the tropical Andes suggests that these populations represent biogeographic “relicts”, and the discovery of *Oryctophileurus guerrai* **sp. n.** in the southern Bolivian Andes suggests that this area is underrated with respect to insect diversity and endemism.

## Abstract (es)

El género *Oryctophileurus* es revisado y su validez confirmada por la combinación de los siguientes caracteres apomórficos: un cuerno cefálico con carina lateral, cavidad pronotal con orificios ocelados y dos dientes o tubérculos detrás del margen anterior pronotal. El macho de *Oryctophileurus varicosus* Prell, 1934, es descrito por primera vez. Una nueva especie *Oryctophileurus guerrai* Perger & Grossi **sp. n.** es descrita del bosque subhúmedo Tucumano-Boliviano en el sureste de los Andes. La nueva especie se distingue por su parentesco más cercano a *O. armicollis* Prell, 1911, por la estrecha distancia entre los dientes interiores de las protuberancias en la región dorsal pronotal y una reducida área con escasos orificios ocelados debajo del margen posterolateral pronotal. Las especies de *Oryctophileurus* ocurren en áreas de endemismo a lo largo de la Vertiente este de los Andes tropicales y sugieren que estas poblaciones representan relictos biogeográficos, y el descubrimiento de *Oryctophileurus guerrai* Perger & Grossi **sp. n.** en el sudeste de los Andes Bolivianos indica que esta área está subestimada en cuanto a la diversidad y endemismo de insectos.

## Introduction

The evolution of exaggerated morphological traits such as cephalic and pronotal horns in male rhinoceros beetles (Dynastinae) has been of interest to biologists for centuries (Rowland and Miller 2012). The Neotropical genera *Oryctophileurus* Kolbe, 1910, and *Amblyodus* Westwood, 1878, belong to the few taxa that lack pronounced gender dimorphism: although less developed, the females bear cephalic horns as well (Grossi and Grossi 2011). However, despite this exceptional feature, both taxa are rarely treated in the scientific literature, presumably because of the difficult accessibility of their forest habitats or a cryptic way of life.

*Oryctophileurus* was established by Kolbe (1910) to stabilize the generic taxonomy of *Phileurus nasicornis* Burmeister, 1847, a species recorded from Colombia. Prell (1911) described a second species, *Oryctophileurus armicollis*, from Peru based on two males. The third species of the genus, *Oryctophileurus varicosus*, was described from a female only without locality record (Prell 1934). Subsequent literature on the taxonomy of *Oryctophileurus* is restricted to redescriptions of the type specimens by Endrödi (1977, 1985) and a cladistic analysis of Phileurini that included *Oryctophileurus armicollis* and *Oryctophileurus varicosus* (Ide 1998).

Ide (1998) recognized a noticeable similarity between *Oryctophileurus* and *Amblyodus*, and Grossi and Grossi (2011) subsequently suggested synonymizing *Oryctophileurus* with the latter. However, the taxonomic position of *Oryctophileurus* has not been revised so far.

The purpose of this contribution is to stabilize the taxonomy of this group through the careful examination of all species originally described in *Oryctophileurus* and *Amblyodus*. A new species of *Oryctophileurus* from the southern Bolivian Andes and the male of *Oryctophileurusvaricosus* are described the first time, and their biogeography is reviewed and briefly discussed.

## Material and methods

We examined 13 specimens deposited in the collection of the Museum für Naturkunde (Humboldt Universität), Berlin, Germany (ZMHB); the Museu de Zoologia, Universidade de São Paulo, São Paulo, Brazil (MZSP); and the Everardo and Paschoal Grossi Private Collection (Nova Friburgo, RJ, Brazil) (EPGC).

**Figure 1.**
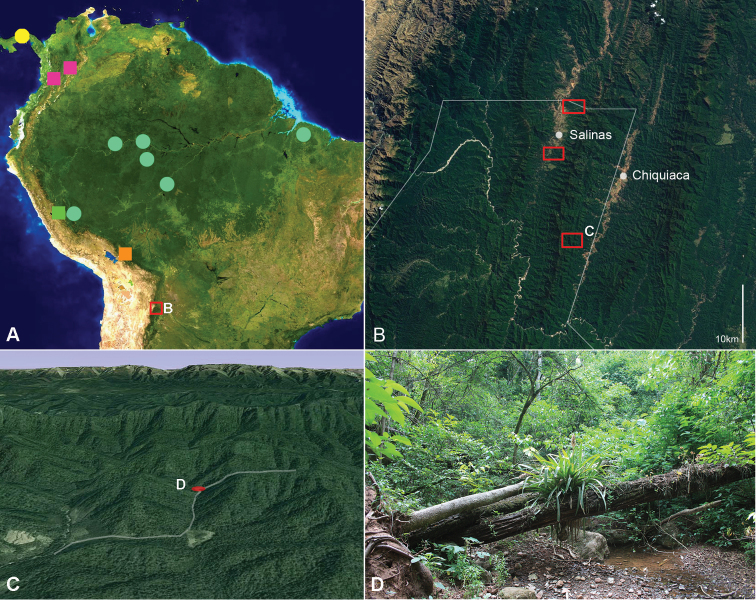
**A** map of distributional locations of South American *Amblyodus* and *Oryctophileurus* species (partly adapted from Grossi and Grossi (2011), only most southern distribution of *Amblyodus taurus* shown), yellow, *Amblyodus taurus*; light blue, *Amblyodus castroi*; magenta, *Oryctophileurus nasicornis*; green, *Oryctophileurus varicosus*; orange, *Oryctophileurus armicollis* (no data for Peru); red, *Oryctophileurus guerrai* **B** Andean and Subandean area of Tarija department, Bolivia; border of Tariquía National Reserve indicated by white line; surveyed areas by red rectangles **C** survey transect (indicated white) with collection location (highlighted red) of *Oryctophileurus guerrai* **D** habitat of *Oryctophileurus guerrai* in the bottom of a moist gully.

Additional specimens were collected during a biodiversity survey headed by the first author in the southern Bolivian Andes, in the northwestern buffer zone of the TariquíaFlora and Fauna National Reserve, department of Tarija, Bolivia. Several transects of about 6 km of subandine, subhumid, semi-deciduous Tucuman-Bolivian forest (Navarro and Ferreira 2011) were surveyed from November to December 2010. The study area and transect images were obtained from Google Earth 2012 and Landsat imagery courtesy of NASA Goddard Space Flight Center and the U.S. Geological Survey (Fig. 1A–C). Collected specimens are deposited in the Colección Boliviana de Fauna (La Paz, Bolivia) (CBF).

**Figure 2.**
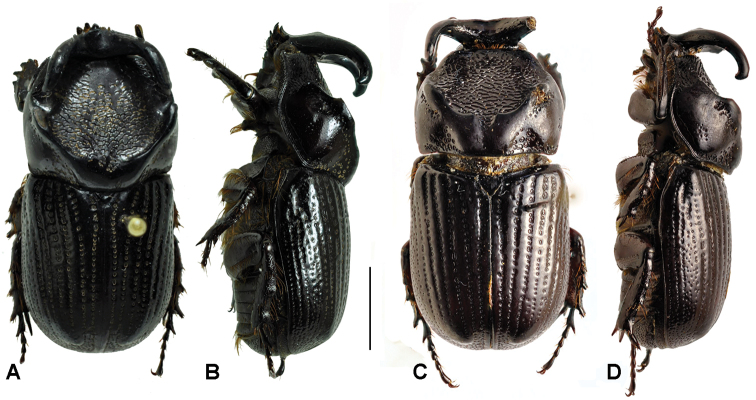
Dorsal and lateralhabitus of males. **A** and **B** *Amblyodus taurus* Westwood, 1878 **C** and **D** *Amblyodus castroi* Grossi & Grossi, 2012, scale bar 5 mm.

Morphological characters were examined with a stereomicroscope, and specimens were sexed via genital dissection. Morphometric measurements were taken with a digital caliper. Body length was measured from the apex of the clypeus to the apex of the pygidium.

A map (Fig. 1A) with the known distribution of treated taxa is included to facilitate the interpretation of biogeographical relationships. Distributional data from the literature is only considered when based on properly identified specimens. Records of *Oryctophileurus* species from Central America (Endrödi 1977; Lachaume 1992) need to be confirmed and are not considered here.

The following abbreviations were used: department, dep.; province, prov.; municipality, muni.

## Systematics

### Oryctophileurus

Kolbe, 1878

http://species-id.net/wiki/Oryctophileurus

#### Type species.

**Figure 3.**
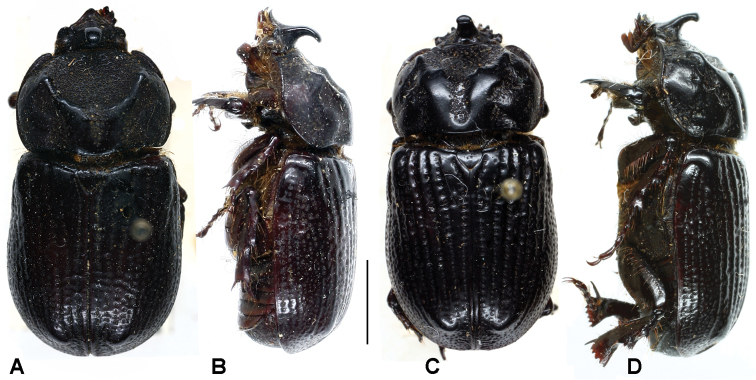
Dorsal and lateral habitus of *Oryctophileurus nasicornis* (Burmeister, 1847) **A** and **B** male **C** and **D** female, scale bar 5 mm.

*Phileurus nasicornis* Burmeister, 1847 (original combination) (Fig. 3).

Species of *Oryctophileurus* are distinguished from other Phileurini by a combination of the following apomorphic characters: a single cephalic horn with lateral carina, pronotal cavity with ocellate punctures and two teeth or tubercles close to the anterior pronotal margin, teeth vertically positioned at about the same level as the outer eye margins.

#### Taxonomy and discussion.

The following characters were cited by Endrödi (1985) for *Oryctophileurus*, *Amblyodus*, *Microphileurus* Kolbe, *Metaphileurus* Kolbe, *Trioplus* Burmeister, and *Goniophileurus* Kolbe and are here not considered as generic: outer side of mandible tridentate; antenna 10-jointed; elytra with punctate rows; proleg sexually monomorphic, protarsus not thickened in male. *Oryctophileurus* and *Amblyodus* are distinguished from the other mentioned genera by large horns, a distinctly developed and posteriorly carinate pronotal cavity and four external teeth on the protibia (Endrödi 1985; Ide 1998). However, in *Amblyodus* the two cephalic horns lack the lateral carina, the punctures in the pronotal cavity are predominately fused to short, transverse ridges, and the teeth close to the anterior pronotal margin are absent. Except for the horn number, these generic differences between *Oryctophileurus* and *Amblyodus* have not been recognized by previous workers.

Based on the combination of apomorphic characters of *Oryctophileurus* that lacks in *Amblyodus* we suggest retaining the genus *Oryctophileurus*.

#### Key to species of *Oryctophileurus*

**Table:** 

1	Horn on frons not projecting above anterior edges of pronotal protuberance (Figs 3B, D). Distance between inner teeth of dorsal pronotal protuberance (Figs 3A, C) narrower than distance between eyes. Development of horn and pronotal protuberance equal in both sexes. Color dark brown	*Oryctophileurus nasicornis* (Burmeister)
–	Horn on frons projecting above anterior edges of pronotal protuberance in both sexes. Horn longer in males than in females of the same size. Color black	2
2	Development of pronotal protuberance subequal in both sexes. Elytral striae and interstitial punctures arranged in irregular lines, punctures enlarged (Figs 4A, C)	*Oryctophileurus varicosus* Prell
–	Pronotal protuberance more strongly developed in males than females of the similar size. Elytral striae and interstitial punctures arranged in regular lines	3
3	Distance between inner teeth of dorsal pronotal protuberance in male wider than width between eyes (Fig. 7A); in females separated by a concavity with ocellate punctures (Fig. 6C). Pronotal concavity accounts for 40–50 % of the dorsal pronotal surface in males. Ocellate punctures above posterior-lateral pronotal margin continuous (Fig. 7C)	*Oryctophileurus armicollis* Prell
–	Distance between inner teeth of dorsal pronotal protuberance in male as wide as width between eyes (Fig. 7B), in females separated by a small fissure only (Fig. 8C). Anterior pronotal concavity accounts for about 30% of the dorsal pronotal surface in males. Area of ocellate punctures above posteriolateral pronotal margin reduced laterally (Fig. 7D), punctures small, shallow and sparse	*Oryctophileurus guerrai* Perger & Grossi, sp. n.

### Oryctophileurus nasicornis

(Burmeister, 1847)

http://species-id.net/wiki/Oryctophileurus_nasicornis

Fig. 3

#### Material examined.

**Colombia:** Cauca dep., Cauca Valley: 1 male with body length 19 mm, det. Endrödi 1976 (ZMHB); Boyacá dep., Muzo muni.: 1 female with body length 20 mm, S. Apollin coll., det. Ohaus, revised by Endrödi (1977) (ZMHB).

#### Diagnosis.

Color dark brown, moderately shining. Horn on frons not projecting above anterior edges of pronotal protuberance, weakly recurved, in female with truncate apex; ocular canthus rounded in male, and subquadrate in female. Pronotum broadly rounded, more in female. Distance between inner teeth of dorsal pronotal protuberance narrower than distance between eyes. Pronotal protuberance on same level with elytra. Development of horn and pronotal protuberance equal in both sexes. Elytra striate, with five well defined discal striae; striae regular, at sides weakly defined; punctures regular, about the same size of those at disc. Meso and metatibiae with 4 to 5 distal teeth. Pygidium regularly convex in both sexes, more in female, and densely and finely wrinkled. Parameres with basal half broad, apex slender, straight.

#### Geographical distribution.

The species is known only from the Cauca Valley in Colombia, which is surrounded by the parallel, peninsula-like projections of the Andean Cordillera Occidental and Cordillera Central and the Muzo municipality which is situated on the eastern slope of the Cordillera Oriental (Fig. 1A). These areas include wet premontane forest and, to a lesser extent, upper montane forests and paramos (Espinal 1992).

### Oryctophileurus varicosus

Prell, 1934

http://species-id.net/wiki/Oryctophileurus_varicosus

Figs 4 ; 5C–D

#### Type material examined.

**Peru:** holotype, female, body length 20.4 mm, unknown locality (ZMHB).

#### Additional material examined.

**Peru:** Junin dep.: Satipo prov., Chanchamayo Valley: 1 male with body length 20 mm (EPGC); Satipo prov., Rio Tambo valley, Paraíso Tuncama, ~1300 m a.s.l.: 1 female with body length 19.4 mm, (EPGC); Rios Pichis & Perene, 600–900 m a.s.l., Soc. Geog. De Lima col., 1 male (MZSP).

#### Diagnosis.

**Figure 4.**
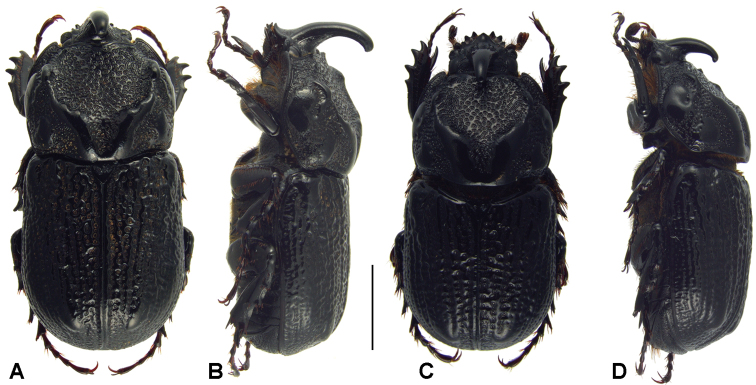
Dorsal and lateral habitus of *Oryctophileurus varicosus* Prell, 1934, **A** and **B** male **C** and **D** female, scale bar 5 mm.

Color black, moderately shining to shining. Enlarged elytral punctures. Punctures and elytral striae arranged in irregular lines (Figs 4A, C). Horn widely projected above body in male. Pronotal protuberance comparably flat and developed as in female.

#### Description.

Male. (Figs 4A, B). Body elongate, cylindrical. Surface entirely black, glabrous, moderately shiny to shiny; dorsally almost totally punctate; punctures ocellate, moderate to very large. Legs, sterna, second abdominal ventrite, and basal margin of pygidium setose.

Head. Surface laterally wrinkled, in frontal view rugose. Clypeus subtriangular, apex rounded and emarginated, weakly reflexed; clypeal carina absent; sides concave. Canthus widely rounded and extending into middle of eye. Frons with long, recurved, cylindrical horn with narrow apex; each side of horn with elongated, weak carina, anterior surface with slightly concave furrow, furrow reaching apex. Mandible tridentate, teeth upturned. Mentum with longitudinal furrow wider anteriorly and posterior concavity narrow with subparallel sides.

Pronotum. Shape subquadrate, narrower than elytra together. Discal area covered by ocellate punctures combined with C-shaped, coalescent punctures; discal surface flat and declivous anteriorly (Fig. 4A, B); anterior and lateral margins complete with a marginal bead, concave at middle; posterior marginal bead absent. Pronotal disc carinate, carina convex, smooth; anterior carina more pronounced; near anterior border with a conspicuous tubercle present in each anterolateral corner; posterior carina joined posteriorly on pronotal margin, bisinuous. Anterior angle acute, posterior rounded. Middle apex laterally with smooth convex carina, intercalated by rugose area and coarse punctures. Prosternal process long, trapezoidal, concave at base and posteriorly produced; base with a spine like posterior process.

Elytra. Striae irregular, and not defined, even laterally; punctures ocellate, irregular, larger on discal area and becoming smaller laterally and posteriorly; elytral apices densely punctate, punctures small to moderate; apical umbone convex, smooth. Scutellum triangular, densely punctate; punctures ocellate, moderate in size. Pygidium. In lateral view widely convex; surface totally punctate; punctures smaller and denser near anterior margin and sides, sparser and larger to apex; apex with marginal bead.

Legs. Protibia with 4 external teeth; basal tooth smaller. Apex of mesotibia with 4 teeth. Apex of metatibia with 5 teeth.

**Figure 5.**
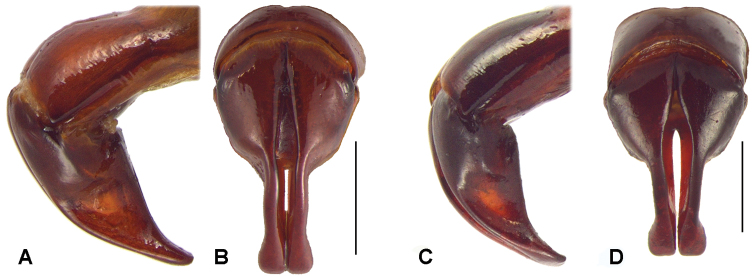
Dorsal and lateral view of aedeagus. **A** and **B** *Oryctophileurusarmicollis* Prell, 1911 **C** and **D** *Oryctophileurus varicosus* Prell, 1934, scale bar 1 mm.

Aedeagus. Shape symmetrical (Fig. 5D), narrowing abruptly at middle; apex inflated, rectangle shaped, truncate; sides subparallel. In lateral view surface concave and with, acute, small projection near lateral base (Fig. 5C).

#### Geographical distribution.

*Oryctophileurus varicosus* was described by Prell (1934) from an unknown locality in Peru. Records from Rio Pichis (600–900 m a.s.l.), Chanchamayo Valley (Junin dep., Satipo prov.) and Paraíso Tuncama (same province) at ~1300 m a.s.l. (Fig. 1A) suggest that this species occurs in the Peruvian Yungas and adjacent subandine transitional forest. The forest in this area is classified as evergreen premontane, subhumid to humid, South Yungas forest (Josse et al. 2003).

#### Remarks.

The records cited here are the only known specimens. Endrödi (1977) described the holotype as a male possibly based upon the fact that it has a horn. Endrödi (1985) correctly redescribed the type specimen as a female, indicating that the male was unknown as was pointed out in the original description by Prell (1934). Ide (1998) cited a male specimen from Rios Pichis & Perene, Peru, which was, however, not described. We describe here the male of *Oryctophileurus varicosus* for the first time.

### Oryctophileurus armicollis

Prell, 1911

http://species-id.net/wiki/Oryctophileurus_armicollis

**Figure 6.**
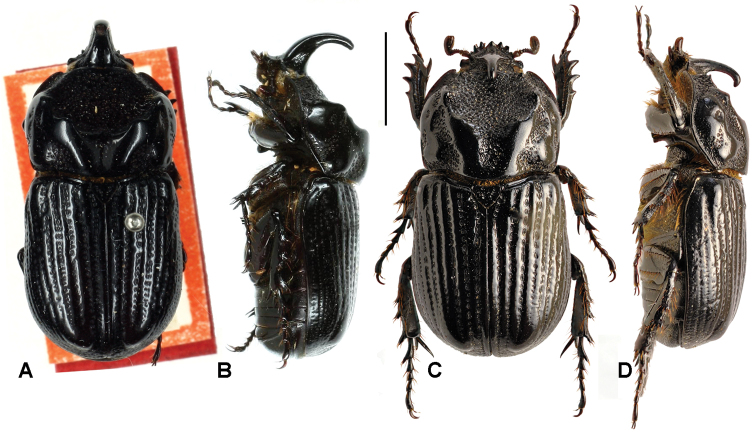
Dorsal and lateral habitus of *Oryctophileurusarmicollis* Prell, 1911, **A** and **B** holotype male, body length 18.5 mm **C** and **D** female, body length 18 mm, scale bar 5 mm.

**Figure 7.**
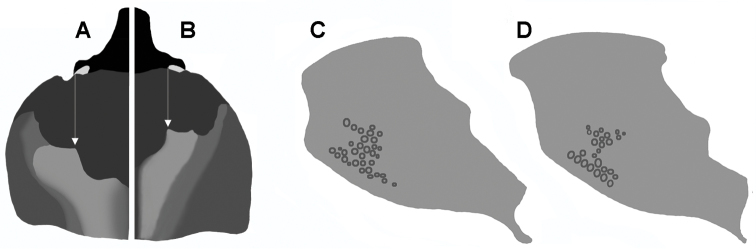
Pronotum of males with body length 20 mm, dorsal, higher areas light, inner teeth of transversal pronotal carina indicated by white arrow:  **A** *Oryctophileurus armicollis* Prell **B** *Oryctophileurus guerrai*; ocellate punctures at posteriolateral pronotal surface, character state does not differ between sexes **C** *Oryctophileurus armicollis* **D** *Oryctophileurus guerrai*.

Figs 5A–B ; 6 ; 7A, C

#### Type material examined.

**Peru:** holotype, male, body length 18.5 mm (ZMHB).

#### Additional material examined.

**Bolivia**: La Paz dep., Nor Yungas prov., Caranavi, I-2003, 2 males with body length 18 mm and 20 mm and 1 female with body length 18 mm (EPGC); Route Coroico-Caranavi, XII-2008, 1 male (EPGC); La Paz dep., Calisaia, V-1925, G.L. Harrington col., 1 male (MZSP); Beni dep., Cosincho, VIII-1925, G.L. Harrington col., 1 female (MZSP).

#### Diagnosis.

Color black, strongly shining. Head anteriorly flat, not concave; canthus subquadrate, in some specimens weakly projected forward; horn in males strongly recurved, on about the same level or slightly higher than posterior pronotal protuberance. Mentum with longitudinal furrow flat and wide, as well as posterior margin. Distance between inner teeth of dorsal pronotal protuberance in male wider that width between eyes (Fig. 6A); in females separated by a concavity with occelate punctures; pronotal concavity accounts for about 40–50 % of dorsal pronotal surface in males. Ocellate punctures above posterior-lateral pronotal margin continuous (Fig. 7C). Each elytron with 10 striae; punctures ocellate, sometimes coalescent and elongated, decreasing in size to sides; interstriae smooth, convex. Striae and interstitial punctures arranged in regular lines Mesotibia with 3 to 5 apical teeth; metatibia with 4 to 6 apical teeth. Pygidium in males distinctly more convex than in females, moderately punctate, denser at base and sides. Aedeagus symmetric (Fig. 5A); parameres with apex rounded to subtriangle shaped (Fig. 5B).

#### Geographical distribution.

*Oryctophileurus armicollis* is known from Peru (Prell 1911; location not specified) and the Andean (Nor Yungas, La Paz dep.) and Subandean (Cosincho, Beni dep.) areas of Bolivia (Fig. 1A). The ecosystem between 800 and 2000 m a.s.l. in this area is considered as South Yungas submontane, subhumid forest (Josse et al. 2003) and receives an annual precipitation between 1500–6000 mm (Ibisch et al. 2003a). The locality data suggests that this species is closely associated with the Bolivian Yungas forest and might also occur in the Peruvian Yungas forest.

### Oryctophileurus guerrai

Perger & Grossi sp. n.

http://zoobank.org/71F1D594-7DC9-41E0-93DE-704DEDE75470

http://species-id.net/wiki/Oryctophileurus_guerrai

Figs 7B, D ; 8

#### Type material.

Holotype: male,“Bolivia / Tarija / O’Connor province / Tariquía National Reserve / S21°59'01, W64°12'30 / 1008 m a.s.l. / Tucuman-Bolivian subhumid forest / gully close to small mountain river / 25-XI-2011 / R. Perger leg.” Allotype: female, same location data as the holotype, 20-XI-2011, F. Guerra leg.

#### Diagnosis.

**Figure 8.**
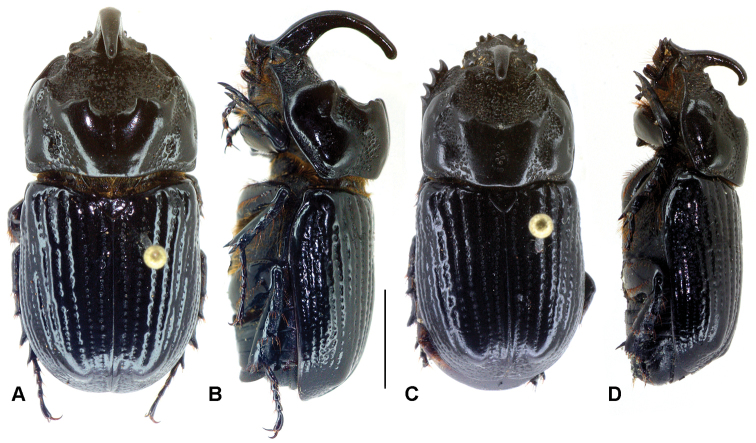
Dorsal and lateral habitus of *Oryctophileurus guerrai* sp. n., **A** and **B** holotype male, body length 20 mm **C** and **D** allotype female, body length 19 mm, scale bar 5 mm.

*Oryctophileurus guerrai* sp. n. is distinguished from the morphologically similar *Oryctophileurus armicollis* by the distance between the inner teeth on the dorsal pronotal protuberance (in males as wide as width between eyes) (Figs 7B, 8A) and in females by the inner teeth separated by only a small fissure (Fig. 8C). In smaller males (body length 18.5 mm) of *Oryctophileurus armicollis* the distance between the inner teeth of the pronotal protuberance is wider than the width between the eyes and in larger males (body length 20 mm) as wide as the distance between the outer eye margins (Fig. 6A). In females of *Oryctophileurus armicollis* the inner teeth of the pronotal protuberance are separated by a comparably wide, continuous, parallel concavity containing ocellate punctures (Fig. 6C).

In both sexes of *Oryctophileurus guerrai* the ocellate punctures above the posteriolateral pronotal margin are smaller, shallower and sparser than in *Oryctophileurus armicollis* and the area of ocellate punctures above posteriolateral pronotal margin is reduced laterally (Figs 7D; 8B, D). In both sexes of other *Oryctophileurus* speciesthe pronotum posteriolaterally has a continuous area of distinctly developed, ocellate punctures.

In the male *Oryctophileurus guerrai* the pronotal protuberance is dorsally higher and the cephalic horn longer (Fig. 8B) than in similar-sized *Oryctophileurus armicollis* (Fig. 6B) and *Oryctophileurus nasicornis* (Fig. 3B). In *Oryctophileurus nasicornis*, both characters are less produced. In the male of *Oryctophileurus varicosus* (Fig. 4B), with its slightly larger body, the pronotum is much flatter than in the male of *Oryctophileurus guerrai*.

#### Description.

Holotype male (Figs 8A, B). Body length 20 mm, width 9 mm. Body elongate, cylindrical, head and pronotum brownish black dorsally, elytra black, body ventrally dark brown, dorsal surface glabrous. Legs, sterna, second abdominal ventrite and basal margin of pygidium setose.

Head. Surface smooth, finely punctate. Clypeus subtriangular, laterally emarginated, slightly upturned, and with acute apex. Cephalic horn recurving over pronotum, attenuate, apex narrowly rounded; surface at base coarsely punctate, with a lateral carina. Mandible tridentate with inner tooth more acute; teeth upturned. Antenna 10 segmented; club with antennomeres subequal in length.

Pronotum (Figs 7B, D; 8A, B). Shape subtrapezoidal, evenly rounded laterally, smooth, with 2 impressions posteriolaterally; posterior edges slightly obtuse. Basal half smooth, longitudinally ridged, dorsally bulging until transverse median carina, with 2 dorsolateral, coarsely punctate concavities on both sides; transverse median carina with 4 obtuse teeth, 2 inner teeth dorsally with small concavity, distance between inner teeth of dorsal pronotal protuberance as wide as width between eyes; anterior half of pronotum strongly concave, with ocellate punctures, 2 obtuse teeth close to anterior pronotal border, teeth at same level with lateral eye margins when seen from dorsal and lateral views. Prosternal process trapezoidal, concave at the base, posteriorly produced; base with spine-like process posteriorly.

Elytra. Surface smooth, with continuous, slightly convex carinae, weakly impressed interstriae, and with ocellate punctures. Pygidium. Shape convex in lateral view; surface densely punctate; punctures ocellate, moderately sized, elongate near basal margin.

Legs. Protibia with 4 teeth, basal tooth weakly developed. Meta- and mesotibia apically with 3 broaden, shovel-shaped teeth, each tooth additionally furnished with small, apical teeth. The specimen was found dead with damaged abdomen, soft parts and genitalia missing.

Female allotype (Figs 8C, D). Similar to male except by the following features: body length 19 mm, width 8.2 mm; head with cephalic horn less developed, reaching only dorsal pronotal protuberances when seen in lateral view; pronotum longer than high, dorsal longitudinal pronotal concavity about as narrow as width of cephalic horn, uppermost teeth of dorsal pronotal protuberances obtuse, separated by a small fissure.

#### Derivation of specific epithet.

The species is named after our friend and colleague, Fernando “Fideo” Guerra, for his lifetime commitment to the investigation of the Bolivian fauna. His participation in the actual survey in the southern Bolivian Andes has led to the discovery and description of several previously unknown taxa (e.g., Perger and Guerra 2012), and he was also the first to collect an individual of *Oryctophileurus guerrai* sp. n.

#### Geographical and ecological distribution.

*Oryctophileurus guerrai* is known only from the northwestern area of Tariquía National Reserve (Tarija department) in the southern Bolivian Andes (Fig. 1). The forest in the this area is considered subandine subhumid, semi-deciduous, Tucuman-Bolivian forest (TBF) (Navarro and Ferreira 2011) with a mean annual temperature of 18.7 °C and an annual rainfall of 1334 mm (SENAMHI 2007). *Oryctophileurus guerrai* is likely endemic to TBF (see discussion below) and might also occur in the northern limit (Santa Cruz department) and the Argentinean portion of this forest type (Jujuy, Salta and Tucuman departments).

The two individuals of the new species were collected in a narrow valley (elevation 1008 m a.s.l.) (Fig. 1C). The female was observed during the day on the floor of the densely vegetated, moist gully (Figs 1C, D). The male was found dead in a similar habitat. No individuals of the species were observed in subhumid forest along the slopes and during eight nights of sampling with a light trap close to the collection area. Like the other species of this genus, which are only known from few individuals, *Oryctophileurus guerrai* might be rare or has a cryptic way of life.

#### Remarks.

As in males of other dynastine taxa with exaggerated secondary sexual traits(e.g., Eberhard 1979; Rowland 2003),the cephalic horn and pronotal structures of *Oryctophileurus armicollis* males (and likely in the males of other *Oryctophileurus* species) vary allometrically. Larger males have larger horns and pronotal armature with respect to their body length. Accordingly, such characters should be compared in specimens having a similar size. Nevertheless, the distance between the inner pronotal protuberance teeth appears to be positively allometric in *Oryctophileurus armicollis*, since it is wider in larger males (body length 20 mm) than in smaller males (body length 18.5 mm). In the male (body length 20 mm) of *Oryctophileurus guerrai* the inner pronotal protuberance teeth are separated by a gap (Fig. 6B) that is narrower than in the smaller male of *Oryctophileurus armicollis* (body length 18.5 mm), indicating that the ratio of body length/ pronotal protuberance teeth distance is never overlapping inter-specifically between similar-sized individuals.

##### Biogeographical affinities

While the two *Amblyodus* species occur in Amazon lowland forest (*Amblyodus castroi* Grossi & Grossi) and Central American mountain forests (*Amblyodus taurus* Westwood) (Grossi and Grossi 2011), the known distributional pattern suggests that the species of *Oryctophileurus* are closely associated with forest habitat in the Andean area (Fig. 1).

As proposed for other Andean taxa (see Hoorn et al. 2010 and Rull 2011 for reviews), the diversification of *Oryctophileurus* might be related to the creation of heterogeneous edaphic mosaics and dispersal barriers by the uplifting of the Andes in the mid-Miocene, marine incursions into the Amazon basin, and the subsequent quaternary climatic cycling. High diversity and endemism in the Andean area are further explained by climatic stability due to orographic rain barriers and lower extinction rates during periods of drastic climatic changes (Fjeldså et al. 1999). The collection locations of *Oryctophileurus* species along the eastern slope of the tropical Andes correspond with peak concentrations of endemics (see Swenson et al. 2012; WWF 2012), suggesting that *Oryctophileurus* species represent biogeographic relicts that persisted during periods of ecological change.

##### *Oryctophileurus* in the Southern Bolivian Andes

The discovery of *Oryctophileurus guerrai* extends the known distributional range of the genus more than 600 km southwards. In view of the mainly tropical Andes distribution of *Oryctophileurus* species, the presence of this genus in the southern Bolivian Andes, close to the Argentinean border, is surprising. Because of a change in orientation of the mountain ranges at the elbow of the Andes and local topographic features, the TBF is distinguished from Bolivian Yungas forest (BYF) by a more pronounced and prolonged dry season, occasionally accompanied by frost periods (Fjeldså et al. 1999), and less annual precipitation and humidity (Ibisch et al. 2003a; Killeen et al. 2007). Corresponding with a general decrease in biodiversity (Schulenberg and Awbrey 1997; Ibisch et al. 2003b; Churchill and Lozano 2009), several scarabaeoid genera such as *Dynastes* Kirby, *Sphaenognathus* Buquet, *Scortizus* Westwood, and *Cantharolethrus* Thomson, meet their southern distributional limit at the elbow of the Andes (see Paulsen 2010 for distributional maps).

Nevertheless, the discovery of *Oryctophileurus guerrai* and other endemic TBF representatives of butterfly genera (Gareca and Blandin 2011; Blandin and Gareca 2011) and tiger beetle genera (Perger and Guerra 2012) with diversity center in the northern tropical Andes suggests that the assumed decrease in species richness in some groups is the result of sampling bias and the TBF belongs to the important areas of insect endemism along the eastern slope of the Andes.

This hypothesis should be tested in further studies because human impact and low protection status of such ecoregion (see Schulenberg and Awbrey 1997; Ibisch et al. 2003a) might not only threat already known but also many undiscovered endemics with extinction.

## Supplementary Material

XML Treatment for Oryctophileurus

XML Treatment for Oryctophileurus nasicornis

XML Treatment for Oryctophileurus varicosus

XML Treatment for Oryctophileurus armicollis

XML Treatment for Oryctophileurus guerrai

## References

[B1] Blandin P Gareca Y (2011) A new subspecies of *Morpho (Grasseia) godartii* Guérin-Méneville, [1844], discovered in sub-humid forests from southern Bolivian Andes (Lepidoptera, Nymphalidae). Bulletin de la Société Entomologique de France 116(3): 291-300.

[B2] Churchill SP Lozano R (2009) Bryophytes of the Tucumán-Bolivian Montane forest. Tropical Bryology 30: 19-42.

[B3] Endrődi S (1977) Monographie der Dynastinae 8. Tribus: Phileurini, amerikanische Arten I. (Coleoptera). Folia Entomologica Hungarica 30(1): 7-45.

[B4] Endrődi S (1985) The Dynastinae of the World. Dr W. Junk, Dordrecht, Netherlands, 800 pp.

[B5] Espinal LS (1992) Geografía ecológica de Antioquia: Zonas de vida. Editorial Léalon, Medellín, Colombia, 146 pp.

[B6] Fjeldså J Lambin E Mertens B (1999) Correlation between endemism and local ecoclimatic stability documented by comparing Andean bird distributions and remotely sensed land surface data. Ecography 22: 63-78. doi: 10.1111/j.1600-0587.1999.tb00455.x

[B7] Gareca Y Blandin P (2011) *Morpho (Morpho) helenor* (Cramer) (Lepidoptera, Nymphalidae, Morphinae) in Bolivia: Geographical distribution and ecological plasticity, with a description of a new subspecies. Zootaxa 3130: 30-56.

[B8] Grossi PC Grossi EJ (2011) A new species of *Amblyodus* Westwood, 1878 (Coleoptera, Melolonthidae, Dynastinae) from South America. ZooKeys 75: 21-28. doi: 10.3897/zookeys.75.884 PMC3088044 21594138

[B9] Hoorn C Wesselingh F Steege HT Mora A Sevink J Sanmartin I Sanchez-Meseguer A Anderson CL Figueiredo J Jaramillo C Riff D Negri FR Hooghiemstra H Lundberg JG Stadler T Sarkinen T Antonelli A (2010) Amazonia through time: Andean uplift, climate change, landscape evolution and biodiversity. Science 330: 927-931. doi: 10.1126/science.1194585 21071659

[B10] Ibisch PL Beck SG Gerkmann B Carretero A (2003a) La diversidad biológica: ecorregiones y ecosistemas. In: Ibisch PL Mérida G (Eds) Biodiversidad: La riqueza de Bolivia. Estado de conocimiento y conservación. Ministerio de Desarrollo Sostenible. Editorial Fundación Amigos de la Naturaleza (FAN), Santa Cruz, Bolivia, 47-88.

[B11] Ibisch PL Gerkmann B Kreft S Beck SG Herzog SK Köhler J Müller R Reichle S Vásquez R (2003b) Consideraciones comparativas de patrones interecoregionales de diversidad de especies y de endemismo. In: Ibisch PL Mérida G (Eds) Biodiversidad: La riqueza de Bolivia. Estado de conocimiento y conservación. Ministerio de Desarrollo Sostenible. Editorial Fundación Amigos de la Naturaleza (FAN), Santa Cruz, Bolivia, 148-161.

[B12] Ide S (1998) Sistemática e evolução dos géneros neotropicais de Phileurini (Coleoptera: Scarabaeidae: Dynastinae). Tese de Doutorado, Universidade de São Paulo, Brasil, 159 pp.

[B13] Josse C Navarro G Comer P Evans R Faber-Langendoen D Fellows M Kittel G Menard S Pyne M Reid M Schulz K Snow K Teague J (2003) Ecological systems of Latin America and the Caribbean: A working classification of terrestrial systems. NatureServe, Arlington, VA, 47 pp.

[B14] Killeen TJ Douglas M Consiglio T Jørgensen PM Mejía J (2007) Dry and wet spots in the Andean hotspot. Journal of Biogeography 34: 1357-1373. doi: 10.1111/j.1365-2699.2006.01682.x

[B15] Kolbe H (1910) Über die Phileurinen Amerikas. Bulletin et Annales de la Societe Royale Belge d’Entomologie 54: 330-354.

[B16] Lachaume G (1992) Dynastidae Américains. Cyclocephalini-Agaocephalini-Pentodontini-Oryctini-Phileurini. Les Coléoptères du Monde 14. Sciences Nat, Venette, France 56: 83–89.

[B17] Navarro G Ferreira W (2011) Mapa de Sistemas Ecológicos de Bolivia, escala 1:250 000. Edición CD-ROM RUMBOL SRL-The Nature Conservancy (TNC), Bolivia.

[B18] Paulsen MJ (2010) Annotated checklist of the New World Lucanidae, version 3.0. http://www-museum.unl.edu/research/entomology/Guide/Scarabaeoidea/Lucanidae/Lucanidae-Catalog/LucanidaeC.htm

[B19] Perger R Guerra F (2012) Two new tiger beetle (Coleoptera, Carabidae, Cicindelitae) species from the Tucuman-Bolivian forest in the National Tariquía Reserve, Bolivia. Zootaxa 3434: 49-58.

[B20] Prell H (1911) Beiträge zur Kenntnis der Dynastinen. Entomologische Zeitschrift 25: 105-107.

[B21] Prell H (1934) Beiträge zur Kenntnis der Dynastinen (XII). Beschreibungen und Bemerkungen. Entomologische Blätter für Biologie und Systematik der Käfer 30: 55-60.

[B22] Rowland JM (2003) Male horn dimorphism, phylogeny and systematics of rhinoceros beetles of the genus *Xylotrupes* (Scarabaeidae, Coleoptera). Australian Journal of Zoology 51: 213–258. doi: 10.1071/ZO02013

[B23] Rowland JM Miller KB (2012) Phylogeny and systematics of the giant rhinoceros Beetles (Scarabaeidae: Dynastini). Insecta Mundi 0263: 1-15.

[B24] Rull V (2011) Neotropical biodiversity: timing and potential drivers. Trends in Ecology & Evolution 26: 508-513. doi: 10.1016/j.tree.2011.05.011 21703715

[B25] Schulenberg T Awbrey K (1997) A rapid assessment of the humid forests of South Central Chuquisaca, Bolivia. RAP Working Papers 8, Conservation International, 84 pp.

[B26] SENAMHI (Servicio Nacional de Meteorología e Hidrología) (2007) Resumen Climatológico 2001–2007 de la Estación Saykan – Las Perulas, Provincia O’Connor, Departamento Tarija, Bolivia, 11 pp.

[B27] Swenson JJ Young BE Beck S Comer P Cordova JH Dyson J Embert D Encarnacion F Ferreira W Franke I Grossman D Hernandez P Herzog SK Josse C Navarro G Pacheco V Stein BA Timana M Tovar A Tovar C Vargas J Zambrana-Torrelio CM (2012) Plant and animal endemism in the eastern Andean slope: Challenges to conservation. BMC Ecology 12(1): 1-18. doi: 10.1186/1472-6785-12-1 22284854 PMC3311091

[B28] World Wildlife Fund (2012) Cauca Valley montane forests. In: Cleveland CJ (Ed) Encyclopedia of Earth. Environmental Information Coalition, National Council for Science and the Environment, Washington, D.C. http://www.eoearth.org/article/Cauca_Valley_montane_forests

